# Screening of potential biomarkers of system lupus erythematosus based on WGCNA and machine learning algorithms

**DOI:** 10.1097/MD.0000000000036243

**Published:** 2023-11-24

**Authors:** Xiaojian Li, Yun Huo, Zhenchang Wang

**Affiliations:** a Guangxi University of Chinese Medicine, Nan Ning, Guangxi, China; b Guangxi International Zhuang Medical Hospital, Nan Ning, Guangxi, China.

**Keywords:** differential gene analysis, immunological infiltration, machine learning algorithms, systemic lupus erythematosus (SLE), weighted correlation network analysis (WGCNA)

## Abstract

Systemic lupus erythematosus (SLE) is an autoimmune disease involving multiple systems. Its recurrent episodes and fluctuating disease courses have a severe impact on patients. Biomarkers to predict disease prognosis and remission are still lacking in SLE. We downloaded the GSE50772 dataset from the Gene Expression Omnibus database and identified differentially expressed genes (DEGs) between SLE and healthy controls. Weighted gene co-expression network analysis was used to identify key gene modules and corresponding genes in SLE. The overlapped genes in DEGs and key modules are used as key genes for subsequent analysis. These key genes were analyzed using 3 machine learning algorithms, including the least absolute shrinkage and selection operator, support vector machine recursive elimination, and random forest algorithms. The overlapped genes were obtained as potential biomarkers for further analysis, investigating and validating the potential biomarkers’ possible functions, regulatory mechanisms, diagnostic value, and expression levels. And finally studied the differences between groups in level of immune cell infiltration and explored the relationship between potential biomarkers and immunity. A total of 234 overlapped genes in DEGs and key modules are used as key genes for subsequent analysis. After taking the intersection of the key genes obtained by 3 algorithms, we got 4 potential biomarkers (ARID2, CYSTM1, DDIT3, and RNASE1) with high diagnostic values. Finally, further immune infiltration analysis showed differences in various immune cells in the SLE and healthy control samples. ARID2, CYSTM1, DDIT3, and RNASE1 can affect the immune function of SLE patients. ARID2, CYSTM1, DDIT3, and RNASE1 could be used as immune-related potential biomarkers and therapeutic or diagnostic targets for further research.

## 1. Introduction

Systemic lupus erythematosus (SLE) is an autoimmune disease. Genetic, immune, endocrine, and environmental factors may all lead to reduced immune tolerance to self-antigens, leading to the formation of pathogenic autoantibodies and tissue damage through a variety of mechanisms.^[1]^ SLE is more common in younger women and involves multiple organ systems, including the musculoskeletal, mucocutaneous, respiratory, digestive, urinary, and hematologic systems.^[2]^ Although about 20% to 30% of SLE patients can enter the chronic active phase of the disease after medical intervention, the majority of patients are still plagued by recurrent episodes and fluctuating disease courses.^[3]^ Due to the insidious onset and complex symptoms of SLE, the diagnosis of SLE patients is often delayed for about 2 years, which will undoubtedly lead to belated treatment.^[4]^ Timely diagnosis and intervention of diseases can help improve the outcome, and organ damage to a certain extent, even reduce the probability of death and recurrence and improve the quality of life.^[5]^ Currently, reliable biomarkers for diagnosing and predicting the onset and progression of SLE are still lacking. Common biomarkers used in clinical practice include the anti-dsDNA antibodies, complement factor proteins, etc., which are limited in diagnosing and monitoring SLE. In the multi-omics era, bioinformatics analysis has theoretically opened the door to studying biomarkers for SLE.^[6]^

With the rapid development of whole-genome sequencing technology and the improvement of public databases and bioinformatics analysis methods, the prediction of disease biomarkers has become more accurate.^[7]^ Some researchers have used bioinformatic analysis from public databases for data mining to promote the screening of new SLE biomarkers.^[8,9]^ Studies combining weighted gene co-expression network analysis (WGCNA) with machine learning algorithms will further improve the accuracy of disease biomarker identification.^[10,11]^ At present, SLE potential biomarkers’ prediction studies are still lack of carried out by combining the 2. Therefore, this study aimed to predict potential biomarkers in the peripheral blood of SLE by combining WGCNA with 3 machine learning algorithms, including least absolute shrinkage and selection operator (LASSO) logistic regression, support vector machine recursive feature elimination (SVM-RFE), and random forest (RF). First, we downloaded the GSE50772 dataset from the Gene Expression Omnibus (GEO) database and identified differentially expressed genes (DEGs) between SLE and healthy controls. In addition, WGCNA was used to identify gene modules and screen for genes associated with SLE progression. The overlapped genes in DEGs and key modules are used as key genes for subsequent analysis. Machine learning algorithms were performed to narrow down the range of potential biomarkers. Subsequently, we investigated the functions of potential biomarkers, their expression levels in different samples, and their diagnostic value for predicting disease conditions and analyzed the correlation between characteristic genes and immunity by comparing the differences in immune cell infiltration between groups. It is hoped that this research can deepen the research on the pathogenesis of SLE, especially the immune-related aspects, provide a more theoretical basis for further elucidating the physiological and pathological mechanisms of SLE occurrence and development, and provide new therapeutic targets for the improvement of patient treatment plans.

## 2. Material and Methods

### 2.1. Downloading and preprocessing of data

We searched the GEO database (https://www.ncbi.nlm.nih.gov/geo/) for SLE gene expression profiles using the keywords “systemic lupus erythematosus” or “SLE.” The datasets are filtered according to the following criteria: First, SLE and healthy control samples must be included in the data set. Second, datasets with peripheral blood mononuclear cells as sequencing samples were preferentially selected. Third, datasets with a sample size >10 cases per group were preferentially selected to improve the accuracy of WGCNA results. Fourth, the selected data set should contain the original data.

### 2.2. DEGs in SLE

The DEGs between the SLE and control group of datasets GSE50772 were identified using the R package “limma.”^[12]^ The cutoff value was |log2(foldchange)| >1, *P* value < .05.

### 2.3. Screening of key modules and genes based on WGCNA

We used the “WGCNA” R package^[13]^ to perform the WGCNA, selecting about 19,000 mean expression >0.5 genes to construct a co-expression network. We performed a hierarchical cluster analysis using the “Hclust” function to exclude outliers samples. Then, an appropriate soft threshold β (1–20) is chosen according to the scale-free network. Subsequently, the neighborhood matrix was transformed into a topological overlap matrix (TOM). Next, we performed hierarchical clustering to identify modules containing at least 30 genes and merged similar modules. Finally, key modules are selected according to the correlation analysis between modules and clinical traits, and the genes in the key modules are selected for key gene screening.

### 2.4. Key genes identification

We used the Venn diagram to identify the overlapped genes in DEGs and key modules.

### 2.5. Functional enrichment analysis of the key genes

Used the “ClusterProfiler”^[14]^ and “DOSE”^[15]^ R packages to perform the biological function enrichment analysis. Gene Ontology (GO) enrichment analysis divides the function of genes into 3 parts: cellular component, molecular function, and biological process. Kyoto Encyclopedia of Genes and Genomes (KEGG) enrichment analysis is used to explore gene-related signaling pathways. Disease Ontology (DO) enrichment analysis is used to enrich related diseases and symptoms. Statistical significance was set at an adjusted *P* value <.05.

### 2.6. Identification of potential biomarkers based on machine learning algorithms

In this study, we used 3 machine learning algorithms to select key potential biomarkers. We applied the LASSO logistic regression algorithm by using the “glmnet” R package in selecting the significant predictive factors.^[16]^ The SVM-RFE algorithm was executed to rank all key genes by using the “e1071” R package.^[17]^ The RF algorithm was performed to feature selection by using the “randomForest” R package.^[18]^ Finally, the intersection of the feature genes obtained by the 3 machine learning algorithms was selected. The overlapping genes obtained were used as potential biomarkers of SLE for subsequent analysis.^[19]^

### 2.7. Assess expression levels and diagnostic significance of potential biomarkers

The Wilcox test was used to calculate the expression level of potential biomarkers in the SLE and healthy control groups. Subsequently, the “pROC” R package was used for receiver operating characteristic (ROC) analysis, and diagnostic value was assessed by area under the curve (AUC).

### 2.8. Analysis of the potential biomarkers using GSEA

Gene set enrichment analysis (GSEA)^[20]^ runs with the “ClusterProfiler”^[14]^ R package. Based on the Pearson correlation between each potential biomarker and other genes, the biological functions of potential biomarkers by the ordered gene expression matrix was investigated.

### 2.9. Immune infiltration analysis by ssGSEA

Using “GSVA” “GSEABase” R packages and “immune. gmt” datasets to investigate the various levels of infiltration of immune cell types between SLE samples and normal samples.^[21]^ A single-sample gene set enrichment analysis (ssGSEA) is a particular type of analysis that combines the “immune. gmt” dataset with the ssGSEA method. It calculates the enrichment score of each sample. With this method, the degree of immune cell infiltration in each sample and the correlations between potential biomarkers expression and the infiltration amount of the immune cells were determined.

## 3. Results

### 3.1. Data collection and workflow

**Figure 1.**
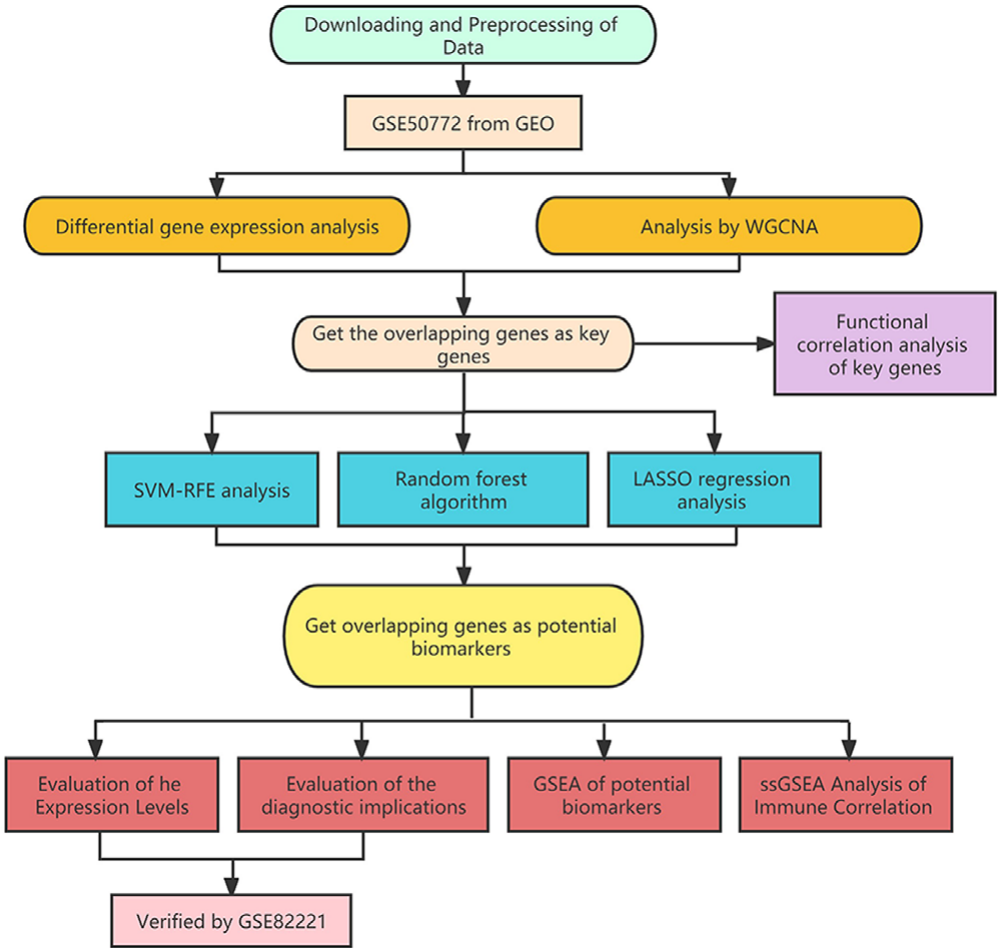
The workflow of the analysis steps.

According to the inclusion and exclusion criteria, we ultimately selected the GEO datasets numbered GSE50772 (GPL570) and GSE82221 (GPL10558). The expression data of 81 samples containing 61 SLE samples and 20 health control was obtained from GSE50772, and the data containing 30 SLE samples and 25 health controls was obtained from GSE82221. We paired the GSE50772 as the discovery cohort and GSE82221 as the validated cohort. The gene symbols of probes were matched according to the corresponding platform annotation files, and the gene expression profiles were normalized. Finally, the dataset is organized into an expression matrix with sample name and gene symbol for subsequent analysis. With the datasets, we further processed the following analysis just shown in the workflow (Fig. 1).

### 3.2. DEGs screening

**Figure 2.**
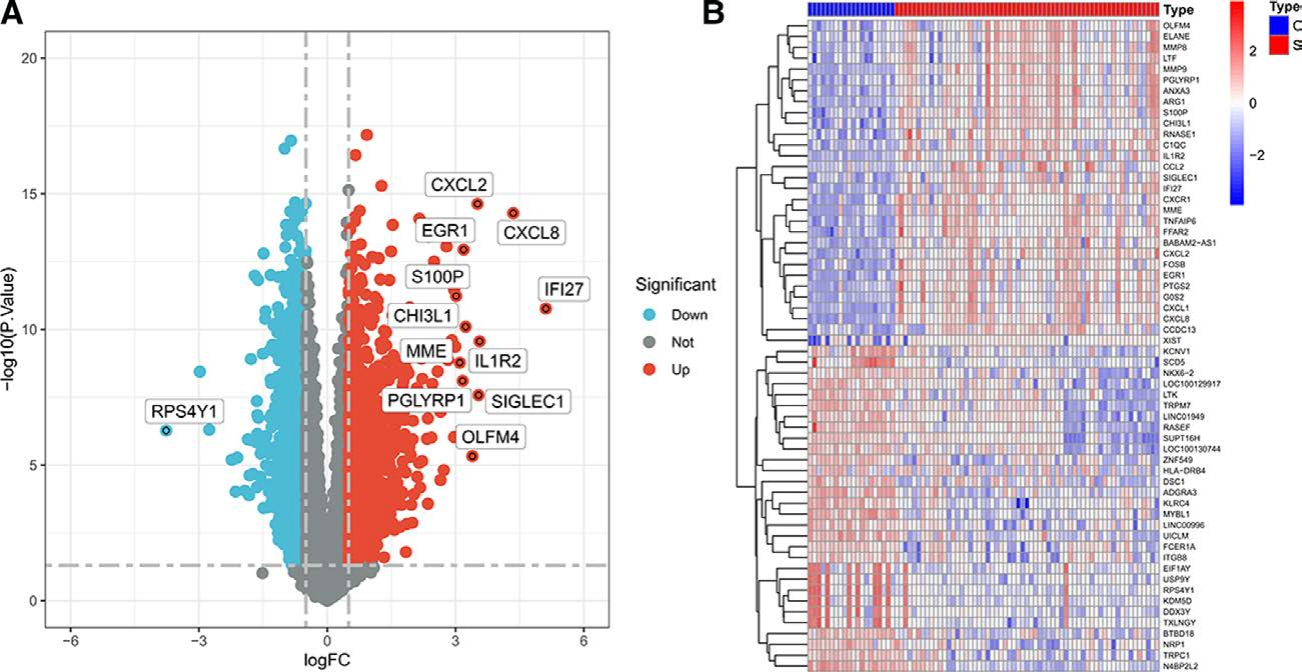
Volcano map and heat map of DEGs in GSE50772. (A) Volcano map in GSE50772. (B) Heat map. 30 DEGs were identified between SLE samples and normal samples. DEGs = differentially expressed genes, SLE = systemic lupus erythematosus.

Under the criteria of |log2(foldchange)| >1, *P* value < .05, a total of 479 genes were identified as DEGs, among which 307 upregulated DEGs and 172 downregulated DEGs (Fig. 2).

### 3.3. Screening of key modules and genes based on WGCNA

**Figure 3.**
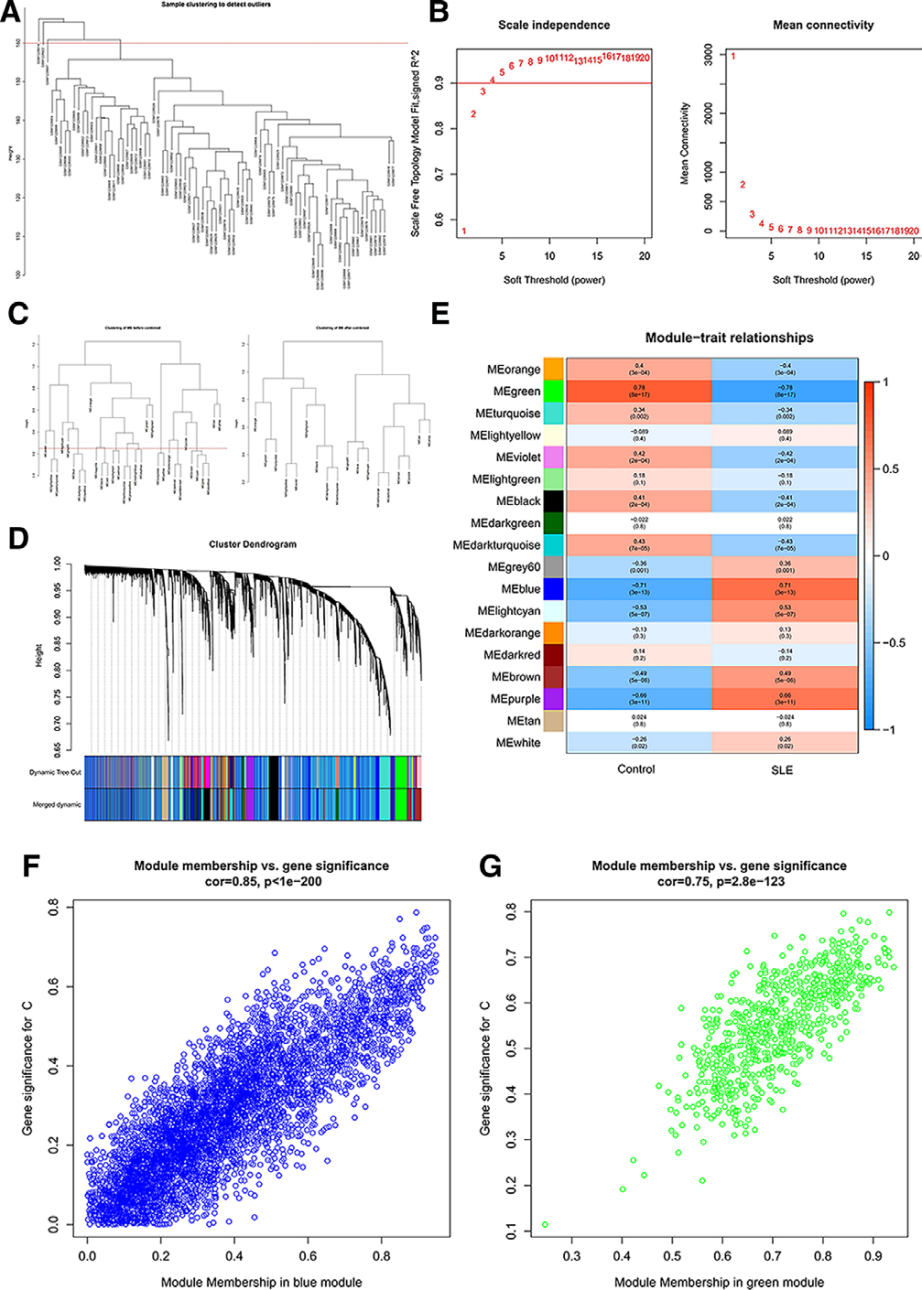
Weighted gene co-expression network analysis. (A) Sample clustering showed 3 outliers were detected by sample clustering. (B) The scale-free fit index and mean connectivity for various soft threshold powers. (C) In hierarchical clustering of the module eigengenes, clustered dendrograms were cut at a height of 0.25 to detect and combine similar modules. (D) The gene cluster dendrogram. Each branch corresponds to a gene, and the color below reflects the different co-expression modules. (E) The correlation between different module eigengenes and traits in a heat map. Red indicates a positive correlation, blue indicates a negative correlation, and a darker color indicates a stronger correlation. (F) MM versus GS scatter plot of the blue module. (G) MM versus GS scatter plot of the green module. GS = gene significance, MM = module members.

After clustering the samples (Fig. 3A), 3 outlier samples were excluded (GSM1228914, GSM1228922, GSM1228897). Network topology analysis was performed for 1 to 20 soft-threshold values, and an optimal soft-threshold value of 4 was chosen (based on the scale-free topology criterion with *R*^2^ > 0.9) (Fig. 3B). Afterward, the weighted adjacency matrix is transformed into a TOM, and the genes are hierarchically clustered according to the topological overlap dissimilarity (diss TOM = 1 − TOM) (Fig. 3C). Then, genes were clustered hierarchically, and a dynamic tree-cutting algorithm cut the dendrogram and obtained 18 modules, using different colors to represent each module (Fig. 3D). Finally, a heat map of module-trait associations is drawn, and the correlation between each module and disease is assessed (Fig. 3E). The blue and green modules were selected as SLE-related modules due to their high correlation with SLE. The green module including 677 genes was correlated positively with health control (*R* = 0.78, *P* = 8e−17) but negatively linked with SLE (*r* = −0.78, *P* = 8e−17), while the blue module including 4974 genes was connected negatively with health control (*r* = −0.71, *P* = 3e−13) and correlated positively with SLE (*R* = 0.71, *P* = 3e−13). Both module members were significantly associated with gene significance (Fig. 3F and G).

### 3.4. Identify key genes and functional correlation analysis

**Figure 4.**
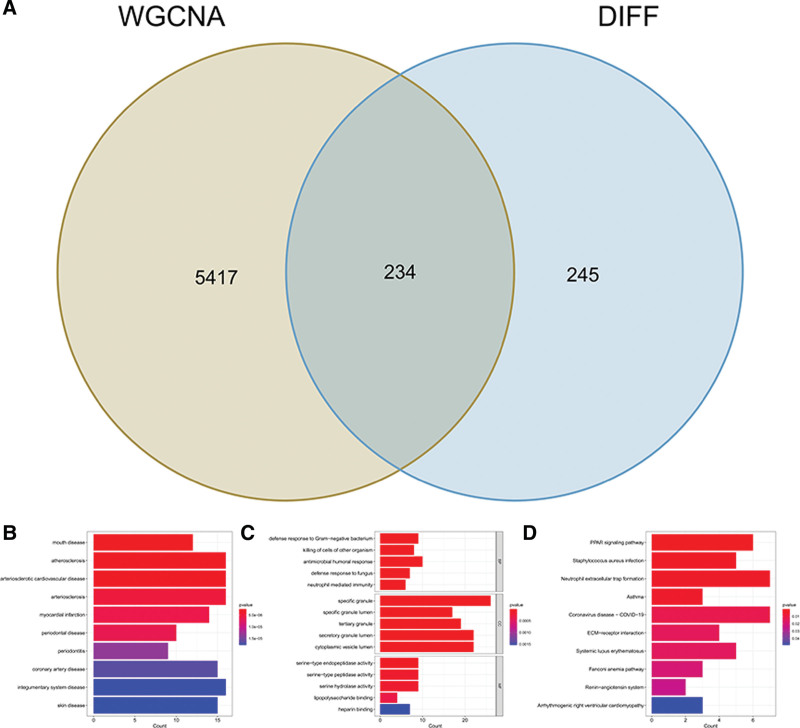
Functional analysis of key genes. (A) Venn diagram of key module genes versus DEGs. (B) The top 10 most significantly enriched DO terms. (C) The top 5 most significantly enriched GO terms. (D) The top 10 most significantly enriched KEGG terms. DEGs = differentially expressed genes, DO = Disease Ontology, GO = Gene Ontology.

Venn diagram analysis revealed 234 overlapping genes between critical module genes and DEGs, and we obtained them as key genes (Fig. 4A). Functional predictions of key genes were achieved by functional enrichment analysis. The DO analysis showed that these key genes were linked to integumentary system disease, arteriosclerotic cardiovascular disease, and mouth disease (Fig. 4B). GO enrichment analysis showed that key genes were mainly enriched in neutrophil-mediated immunity, killing of cells of another organism, defense response to gram-negative bacterium, cytoplasmic vesicle lumen, secretory granule lumen, tertiary granule, specific granule, heparin-binding, lipopolysaccharide-binding, and serine hydrolase activity (Fig. 4C). KEGG cluster analysis showed that the renin–angiotensin system, Fanconi anemia pathway, SLE, ECM–receptor interaction, neutrophil extracellular trap formation, and peroxisome proliferator activated receptor signaling pathway were significantly enriched (Fig. 4D).

### 3.5. Selection of potential biomarkers

**Figure 5.**
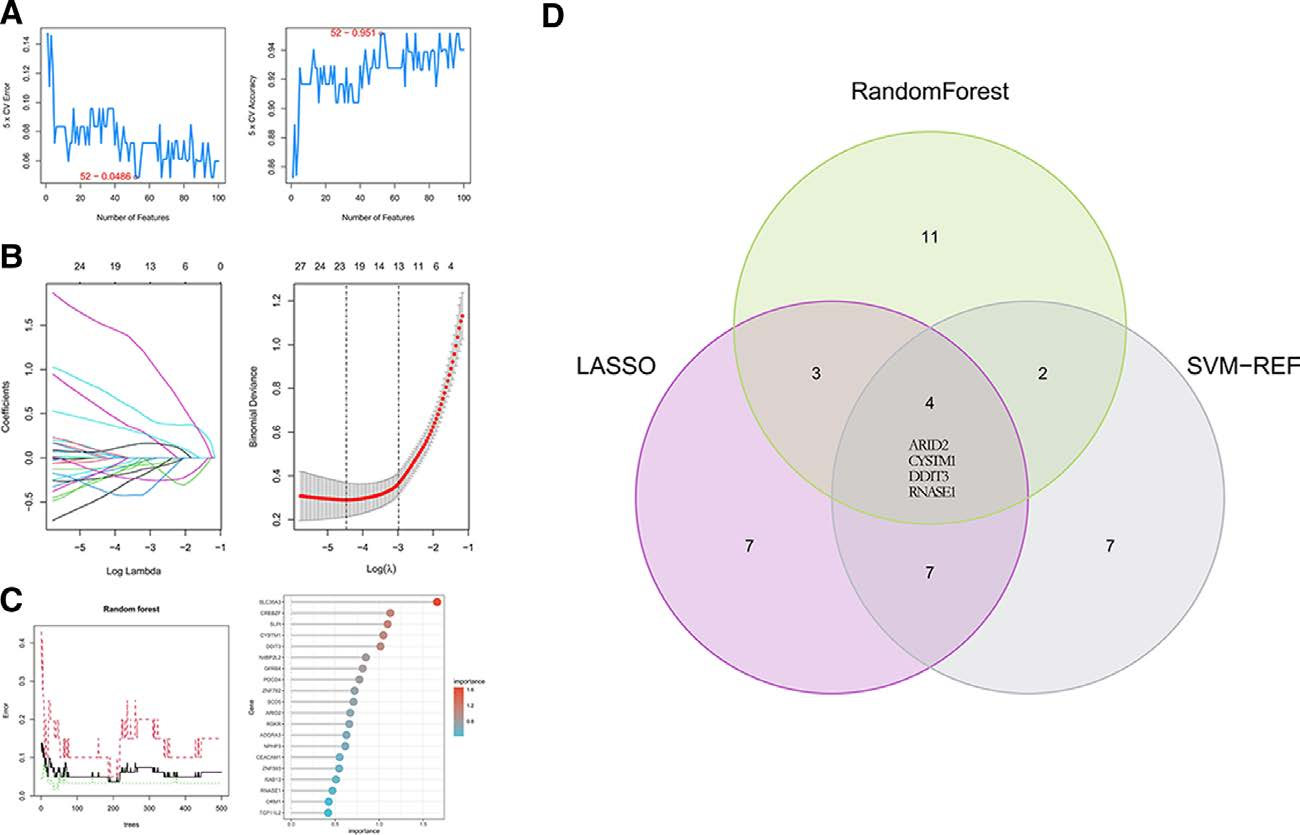
Potential biomarkers selection. (A) Predicted genes by SVM-RFE algorithm selection. (B) Predicted genes selection in LASSO. (C) The relationship between the error rate, the number of classification trees, and the top 20 relatively important genes. (D) Three algorithmic Venn diagram screening potential biomarkers. LASSO = least absolute shrinkage and selection operator, SVM-RFE = support vector machine recursive feature elimination.

Three machine learning algorithms were employed to select potential biomarkers. First, SVM-RFE analysis results show that the SVM model based on 52 key genes showed an optimum error rate (0.0486) (Fig. 5A), and the top 20 key genes were selected as the predicted genes according to average rank. On the other hand, 21 predictive genes with non-zero coefficients were screened by LASSO regression analysis (Fig. 5B). At the same time, the importance of key genes was calculated in RF feature selection, and the 20 genes with the highest priority were considered the predicted genes (Fig. 5C). The Venn diagram was used to take the intersection of the predicted genes obtained by the above 3 methods. Four overlapping genes (ARID2, CYSTM1, DDIT3, RNASE1) were finally obtained as potential biomarkers for further analysis (Fig. 5D).

### 3.6. Evaluation and validation of the expression levels and diagnostic implications for the potential biomarkers

**Figure 6.**
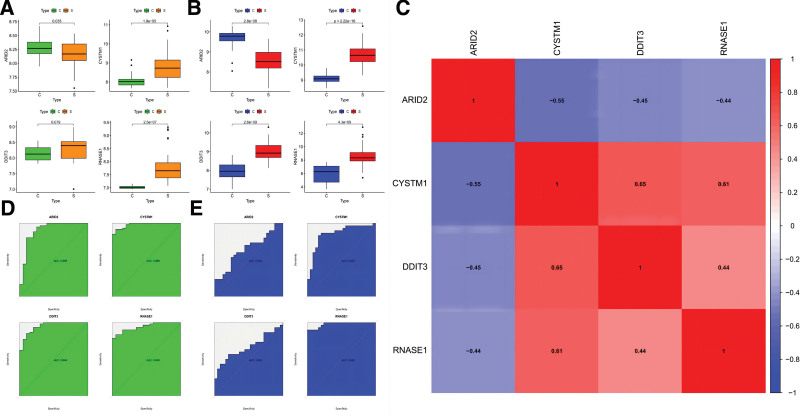
The expression levels and diagnostic implications of the potential biomarkers. (A) The expression levels of potential biomarkers in the discovery cohort. ARID2 was lowly expressed in SLE. CYSTM1, DDIT3, and RNASE1 were highly expressed in SLE. (B) The expression levels of potential biomarkers in the validated cohort. ARID2 was lowly expressed in SLE, but the difference was not statistically significant (*P* > .05); CYSTM1, DDIT3, and RNASE1 were highly expressed in SLE. (C) Correlation between potential biomarkers. (D) ROC curves of potential biomarkers in the discovery cohort. (E) ROC curves of potential biomarkers in the validated cohort. ROC = receiver operating characteristic, SLE = systemic lupus erythematosus.

To further explore the roles of ARID2, CYSTM1, DDIT3, and RNASE1 in SLE, we first investigated the differences in their expression levels between SLE samples and healthy controls. We found that the expression of ARID2 was downregulated in SLE samples compared with the control sample. CYSTM1, DDIT3, and RNASE1 were all substantially elevated in the SLE samples (Fig. 6A). To further understand the accuracy and reliability of the above results, we used the GSE82221 dataset to verify the expression levels of 4 potential biomarkers, among which the expression levels of CYSTM1, DDIT3, and RNASE1 were consistent with the previous results. The expression level of ARID2 in the GSE82221 dataset was also higher in the control group than in the SLE group, but the difference was not statistically significant (*P* > .05) (Fig. 6B). Figure 6C shows gene correlations; CYSTM1, DDIT3, and RNASE1 were positively correlated, and ARID2 was negatively correlated with the other 3 potential biomarkers (Fig. 6C). ROC analysis was performed to evaluate the diagnostic value of potential biomarkers. ARID2 (AUC: 0.899), CYSTM1 (AUC: 0.980), DDIT3 (AUC: 0.948), and RNASE1 (AUC: 0.943) might be used as diagnostic biomarkers of SLE patients (Fig. 6D). The validation datasets (GSE82221) also corroborated the diagnostic value of potential biomarkers: ARID2 (AUC: 0.644), CYSTM1 (AUC: 0.853), DDIT3 (AUC: 0.664), and RNASE1 (AUC: 0.980) (Fig. 6E).

### 3.7. Biological functions of the potential biomarkers

**Figure 7.**
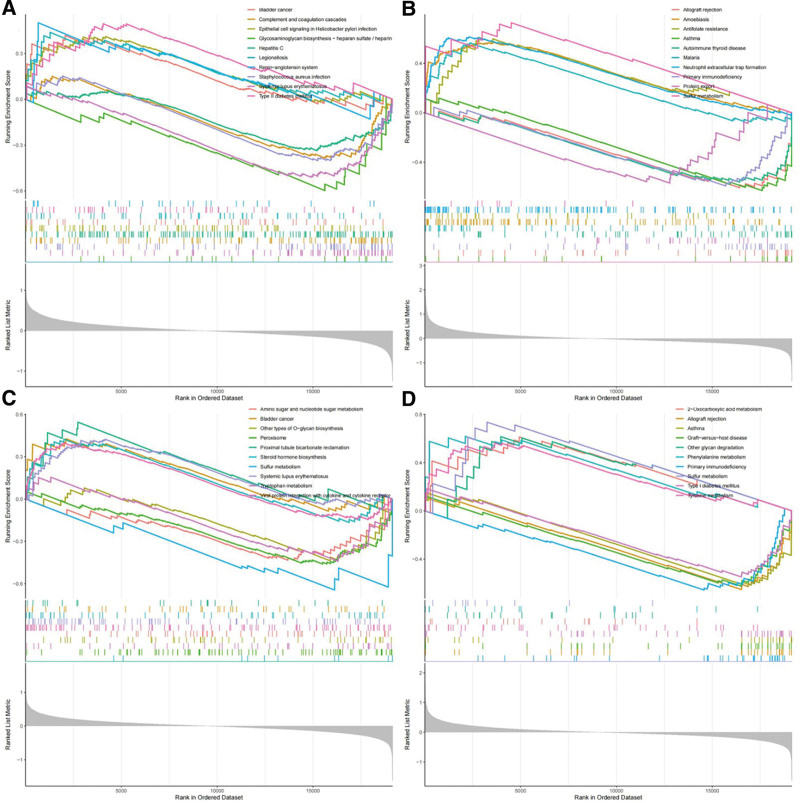
GSEA of potential biomarkers. (A) KEGG enrichment results for ARID2. (B) KEGG enrichment results for CYSTM1. (C) KEGG enrichment results for DDIT3. (D) KEGG enrichment results for RNASE1. GSEA = gene set enrichment analysis, KEGG, Kyoto Encyclopedia of Genes and Genomes.

To further investigate the biological functions and regulatory pathways of ARID2, CYSTM1, DDIT3, and RNASE1, we also performed enrichment analyses using the KEGG pathway dataset from GSEA. The ARID2 high-subgroup was significantly enriched in bladder cancer, epithelial cell signaling in *Helicobacter pylori* infection, Legionellosis, renin–angiotensin system, and Type II diabetes mellitus, yet complement and coagulation cascades, glycosaminoglycan biosynthesis − heparan sulfate/heparin, hepatitis C, *Staphylococcus aureus* infection, and SLE had significant enrichment in the low ARID2 subgroup (Fig. 7A). Amoebiasis, antifolate resistance, malaria, neutrophil extracellular trap formation, and sulfur metabolism had significant enrichment in the high CYSTM1 subgroup. In contrast, allograft rejection, asthma, autoimmune thyroid disease, primary immunodeficiency, and protein export had significant enrichment in the low CYSTM1 subgroup (Fig. 7B). Bladder cancer, proximal tubule bicarbonate reclamation, steroid hormone biosynthesis, SLE, and viral protein interaction with cytokine and cytokine receptors had significant enrichment in the high DDIT3 subgroup. However, amino sugar and nucleotide sugar metabolism, other types of O-glycan biosynthesis, peroxisome, sulfur metabolism, and tryptophan metabolism had significant enrichment in the low DDIT3 subgroup (Fig. 7C). 2-Oxocarboxylic acid metabolism, other glycan degradation, phenylalanine metabolism, sulfur metabolism, and tyrosine metabolism had significant enrichment in the high RNASE1 subgroup. In contrast, allograft rejection, asthma, graft versus host disease, primary immunodeficiency, and type I diabetes mellitus significantly enriched the low RNASE1 subgroup (Fig. 7D).

### 3.8. Immunological infiltration in the SLE sample and healthy controls based on ssGSEA analysis of immune correlation

**Figure 8.**
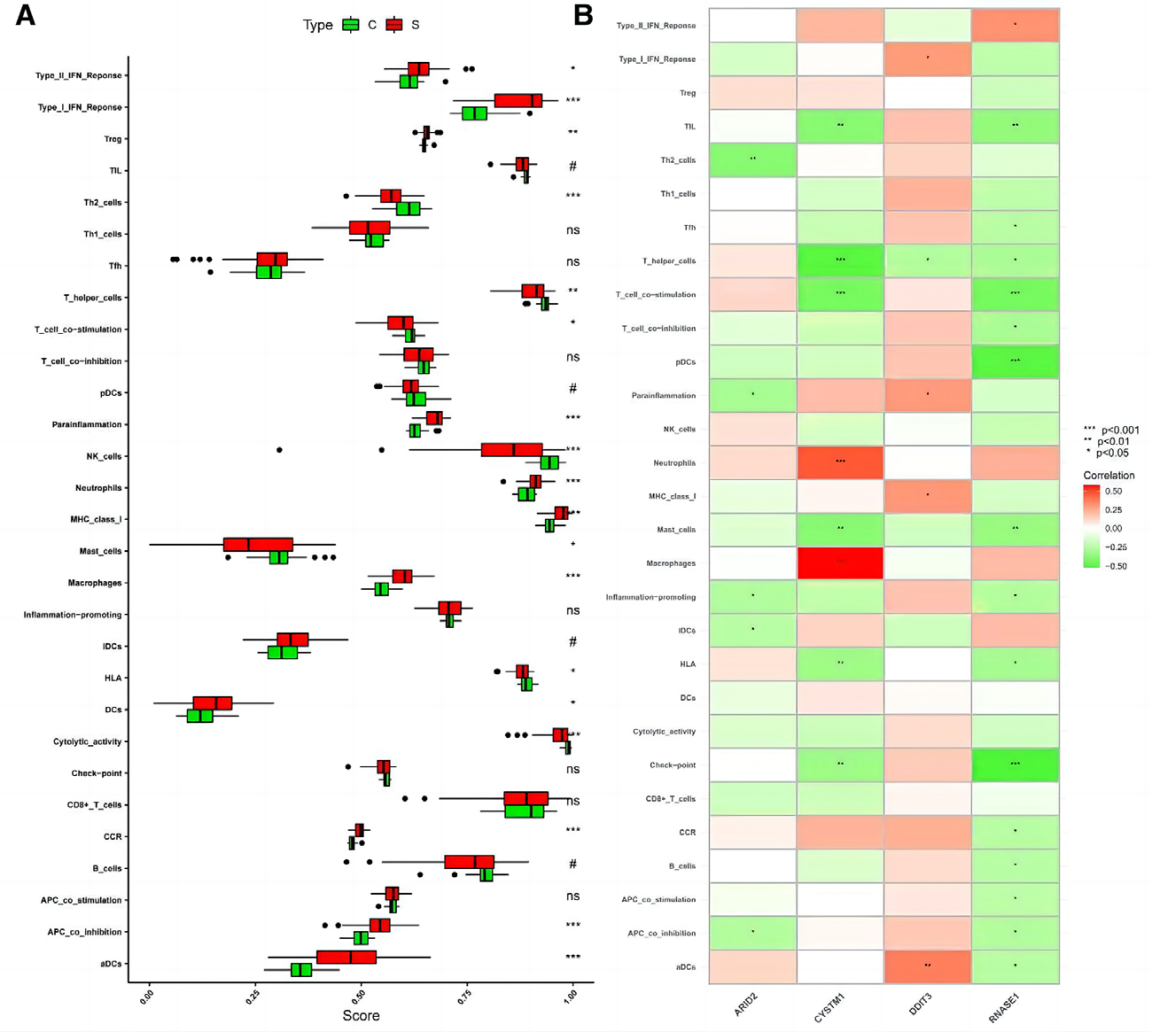
Correlation between SLE and immunity. (A) Comparison of ssGSEA scores between SLE group and healthy controls: scores of immune cells and immune pathways. (B) Correlation between potential biomarkers and immunity (ns represents .2 < *P* ≤ 1, # represents .05 ≤ *P* ≤ .2, * represents .01 ≤ *P* < .05, ** represents 0.01 ≤ *P* < .01, *** represents *P* = 0). SLE = systemic lupus erythematosus, ssGSEA = single-sample gene set enrichment analysis.

To investigate the immune infiltration association between SLE samples and healthy controls, ssGSEA was employed. After excluding nonstatistical significance types, immune cell infiltration of T helper cells, Th2 cells, NK cells, human leukocyte antigen (HLA), and cytolytic activity was lower in SLE than in controls. The SLE group’s other immune cell types and immune-related features had higher immune infiltration than the control group (Fig. 8A). To understand the relationship between potential biomarkers and the immune composition of SLE, we studied the association between expression levels and different immune infiltration. ARID2 was negatively correlated with Th2 cells, parainflammation, inflammation-promoting, iDCs, and APC co-inhibition. CYSTM1 was negatively correlated with neutrophils, macrophages, TIL, T helper cells, T cell co-stimulation, mast cells, HLA, and checkpoint all had positive correlations with CYSTM1. DDIT3 was positively correlated with type I IFN response, ads, MHC class I, and parainflammation and related to T helper cells in a negative way. RNASE1 was positively correlated with type II IFN response and related to B cells, APC co-stimulation, checkpoint, HLA, mast cells, APC co-inhibition, T cell co-stimulation, T cell co-inhibition, T helper cell, TIL, CCR, and pDCs in a negative way (Fig. 8B).

## 4. Discussion

SLE is an autoimmune disease that often occurs in young women and may lead to multiple organ damage. Its occurrence and development involve numerous signal pathways, symptoms are variable, and easy to relapse. In human immune profiling studies, helped by recent technological advances, especially in single-cell and “omics” analyses, enormous progress has been made in the immunological and genetic understanding of SLE. Currently, various targeted therapy regimens involving IFN, cytokines, chemokines, and B cells are undergoing clinical trials. With the increase in treatment options, the identification of biomarkers has become particularly urgent; The biomarkers will improve and guide future clinical management of SLE.^[22]^ However, no validated biological biomarker can predict disease course and treatment response with high reliability and reproducibility. Given these, we used a variety of bioinformatics analysis and machine learning algorithms to predict potential SLE biomarkers, aiming to provide potential new targets and new directions for clinical studies and treatment for early detection. In this study, 307 genes are significantly upregulated, and 172 are downregulated. WGCNA analysis showed 78 cluster samples and 18 modules. Two of the modules were considered to have a significant correlation with SLE. The 2 modules contain a total of 5651 genes. By drawing a Venn diagram, the DEGs were intersected with 5651 genes in 2 modules, and 234 overlapping genes were obtained as key genes for further study. GO enrichment analysis of key genes showed significant enrichment of GO terms associated with serine and neutrophil-mediated immunity. KEGG enrichment analysis based on key genes showed that most KEGG pathways were enriched in SLE, anemia, and peroxisome proliferator activated receptor signaling pathways. Furthermore, DO pathway enrichment analyses revealed that enriched symptoms were predominantly related to skin disease, mouth disease, and arteriosclerosis.

This is consistent with the characteristics of SLE causing multi-system damage. At the same time, recent studies have found that the leading cause of death among SLE patients is a cardiovascular disease driven by accelerated atherosclerosis, and many of the immune mechanisms that drive SLE also promote atherosclerosis.^[23]^

We then identified ARID2, CYSTM1, DDIT3, and RNASE1 as potential biomarkers with 3 machine algorithms, the LASSO logistic regression, SVM-RFE, and RF algorithms. Both the discovery cohort and validated cohort confirmed that ARID2 showed low expression in SLE samples, and CYSTM1, DDIT3, and RNASE1 is just the opposite. To further illustrate the diagnostic role of potential biomarkers in SLE, we performed ROC analysis on the above 4 potential biomarkers. The results of both the discovery cohort and validated cohort showed that all potential biomarkers could better distinguish SLE samples and healthy control samples; this result proves to some extent that the biomarkers have potential diagnostic value. ARID2 has been mutated in various cancers, including hepatocellular carcinoma, malignant mesothelioma, pancreatic cancer, primary clear cell adenocarcinoma of the urethra, microsatellite unstable colon cancer, non-small cell lung cancer, melanoma, and pancreatic cancer.^[24–28]^ CYSTM1 is a relatively unknown gene that has been shown to play a role in stress responses and confer tolerance to heavy metals such as cadmium and copper.^[29]^ Bioinformatics analysis suggests that it is likely to be a biomarker for Huntington disease.^[30]^ DDIT3 encodes a nuclear protein of the enhancer-binding protein family and performs many biological functions, including cell survival and endoplasmic reticulum stress apoptosis and adipogenesis.^[31]^ DDIT3 promotes and restricts lipoblasts and liposarcoma morphology. However, the role in the pathogenesis of DDIT3 in SLE still needs further research. At the same time, research on the RNASE1 gene in the SLE field is also lacking. RNASE1 belongs to the ribonuclease A superfamily of which 13 enzymes have been described.^[32]^ In recent years, a series of studies have proved that the changes of the RNase1-eRNA system are closely related to the occurrence of various vascular diseases such as atherosclerosis and thrombosis.^[33,34]^

Most of the current research related to RNASE1-Erna focuses on the effects on cardiovascular and cerebrovascular. Still, there are reports in the literature that RNASE1 also plays an essential role in other diseases, such as inflammation and infectious diseases.^[35,36]^ There is still no definitive study showing the direct impact of the above 4 biomarkers in the pathogenesis and progression of SLE. Thus, further investigations are necessary.

To further explore the signaling pathways of potential SLE biomarkers, we performed a GSEA analysis. We found that the high CYSTM1 subgroup, high RNASE1 subgroup, and low DDIT3 subgroup were significantly enriched in sulfur metabolism. Primary immunodeficiency was enriched in subgroups with low expression of CYSTM1 and low expression of RNASE1. The low-expression ARID2 and the high-expression DDIT3 groups were enriched in SLE group. Studies have shown that these signaling pathways are directly or indirectly associated with the progression of SLE.^[37]^ Sulfur and sulfur metabolism are correlated with redox reactions, suggesting that redox reactions may be involved in the pathological changes of SLE.

Immunodeficiency can be divided into primary and secondary. Primary immunodeficiency is associated with various genetic diseases of the immune system. These genetic mutations that affect the development, differentiation, and function of immune cells will lead to the occurrence of immunodeficiency. The corresponding secondary immune deficiency is associated with immune system dysfunction caused by many external factors, including the influence of immunosuppressive drugs, malignant tumors, and chronic diseases. Although SLE and immune deficiency are 2 distinct disease entities, like 2 sides of the same coin, SLE patients are susceptible to infection due to multiple reasons, such as the simultaneous involvement of SLE susceptibility genes in the pathogenesis of immune deficiency, the immune dysfunction of SLE patients, and the immunosuppressive effect of SLE treatment drugs.^[38,39]^ Finally, further immune infiltration analysis revealed differences in innate and adaptive immune cells between SLE samples and healthy control samples, including dendritic cells, type 1 IFN response, monocytes and macrophages, neutrophils, T-cell subsets, MHC class I, and NK cells. The influence of immune dysregulation, including innate and adaptive immunity, on SLE, has been widely studied. SLE is a multi-factor and complex autoimmune disease, and researchers have tried to study its pathogenesis from multiple aspects. The dysregulation of the immune response has been widely studied.

From the perspective of innate immunity, dendritic cells are one of the most efficient antigen-presenting cells; they can promote the activation of primitive T cells and stimulate the proliferation of B cells, in addition to playing a role in innate immune, but also have an impact on the adaptive immune system. Type I IFN have many different effects on the immune system, promoting and maintaining autoreactive immune responses. Studies have shown that patients with SLE have elevated serum IFN-α levels, which correlate with disease activity and severity,^[40]^ and biologics targeting type I IFN bring new possibilities for the treatment of SLE.^[41]^ Early studies have found that the ability of macrophages to clear apoptotic cell debris in SLE patients is defective,^[42]^ and recent studies have shown that abnormal activation or interference in the coordination of immune response between monocytes and macrophages will lead to and aggravate immune dysregulation.^[43]^ Neutrophils are involved in the process of inflammation and infection and are an important component of the innate immune system.

When neutrophils become abnormal, there will be an abnormal release of proteases, tissue damage factors, and reactive oxygen species, causing secondary damage to SLE patients. In addition, a variety of cytokines and chemokines released by activated neutrophils will further lead to the disorder of immune regulation.^[44]^ From the perspective of adaptive immunity, disruption of immune tolerance by T cells contributes to the progression of SLE. The imbalance of T-cell subsets, including Th1, Th2, and Treg cells, is involved in the developmental mechanism of SLE.^[45]^ Immunological pathogenesis has a pivotal function in the pathogenesis of SLE.

Innate and adaptive immunity affect each other, and their relationship network is closely connected and complement each other. None of them can exist independently. In addition, cytokines, complement, immune complexes, and kinases of the intracellular machinery play essential roles in SLE, and many trials about it are ongoing. Our results, combined with previous studies, suggest that ARID2, CYSTM1, DDIT3, and RNASE1 may affect the immune function of SLE patients. Still, the mechanisms involved are complicated, and interaction requires further experiments to verify, which is also the shortcoming of this study.

## 5. Conclusions

ARID2, CYSTM1, DDIT3, and RNASE1 expressions were changed in the peripheral blood mononuclear cells of the SLE sample, highly suggested for being used as the potential biomarkers with SLE to further research. The in-depth study of these 4 potential biomarkers may provide novel insights into the pathogenesis of SLE. Furthermore, its association with immune cell infiltration may facilitate the development of immunotherapies for SLE. Further studies are needed to validate and develop experimental research for these potential biomarkers.

## Author contributions

**Conceptualization:** Xiaojian Li.

**Data curation:** Xiaojian Li, Yun Huo.

**Formal analysis:** Zhenchang Wang.

**Funding acquisition:** Zhenchang Wang.

**Investigation:** Zhenchang Wang.

**Resources:** Xiaojian Li, Yun Huo.

**Software:** Yun Huo.

**Supervision:** Zhenchang Wang.

**Validation:** Yun Huo.

**Visualization:** Yun Huo.

**Writing – review & editing:** Zhenchang Wang.

**Writing—original draft:** Xiaojian Li.
